# Orbital and medial rectus muscle involvement as initial presentations of hydatid disease

**DOI:** 10.1186/s12348-025-00476-8

**Published:** 2025-03-07

**Authors:** Seyed Mohsen Rafizadeh, Amir Mousavi, Mohammad Taher Rajabi, Amirhossein Aghajani, Zohreh Nozarian, Amin Zand

**Affiliations:** 1 Department of Oculo-Facial Plastic and Reconstructive Surgery, Farabi Eye Hospital, Tehran University of Medical Sciences, Tehran, Iran; 2 Department of Pathology, Farabi Eye Hospital, Tehran University of Medical Sciences, Tehran, Iran

**Keywords:** Hydatid cyst, Hydatid disease, Orbital cyst, Echinococcosis

## Abstract

**Purpose:**

To report a rare case of orbital hydatid cyst involving the medial rectus muscle, which presented as progressive proptosis, with subsequent detection of liver involvement after further investigations.

**Case presentation:**

We present the case of a 12-year-old boy from a rural area with exposure to wildlife dogs. The patient had a two-month history of gradually progressive proptosis in the right eye, accompanied by periorbital swelling and limited medial ocular motility. Orbital magnetic resonance imaging (MRI) revealed a large mass within the medial rectus muscle, which showed peripheral enhancement with no central enhancement, consistent with a cystic lesion based on its imaging characteristics. The patient underwent orbitotomy, during which the lesion was aspirated, and its walls were resected. Pathological examination confirmed a structure of a hydatid cyst. Given the suggestive signs of a hydatid cyst as part of a systemic echinococcal infection, further investigations, including liver sonography, revealed a similar cystic lesion in the hepatic lobe. With the diagnosis of an orbital hydatid cyst and suspected echinococcal infection, the patient was treated with oral Albendazole for one month. His symptoms, including periorbital swelling, improved, and no recurrence was observed at a six-month follow-up.

**Conclusions:**

Orbital hydatid cysts may present as inflammatory proptosis and should be considered in populations from endemic areas of human echinococcosis. Early diagnosis using orbital MRI, systemic investigations such as liver sonography, timely surgery for definitive diagnosis and treatment, and appropriate adjuvant antiparasitic medication are crucial for effective management.

## Introduction

Hydatid disease is a zoonotic infection caused by the larval stage of the tapeworm *Echinococcus granulosus* [1]. Humans are accidental intermediate hosts, infected through the ingestion of eggs. The hydatid cyst most commonly affects the liver (50–70% of cases), followed by the lungs, spleen, kidneys, and brain [2–4]. Orbital hydatid cysts are rare, representing approximately 1–2% of all hydatid disease cases [5–7]. They may present with various symptoms, including periorbital swelling, eye injection, proptosis, or eye deviation [7]. Due to the rarity of this condition and the need for timely diagnosis and treatment to prevent ocular and systemic complications, ophthalmologists should remain vigilant, especially in endemic areas.

While orbital involvement of hydatid disease has been reported, there are few cases of orbital presentations with undiagnosed systemic involvement [8]. In this report, we aim to present a case of systemic hydatid disease that initially manifested in the orbit, with liver involvement detected upon further investigation.

## Case report

This case study adheres to the CARE guidelines [9]. All procedures were conducted in accordance with the principles outlined in the Declaration of Helsinki. Written informed consent for the publication of the report and related images was obtained from the parent of the patient, and all patient details were de-identified. Ethical approval for case reports was not required by the institutional review board.

**Fig. 1:**
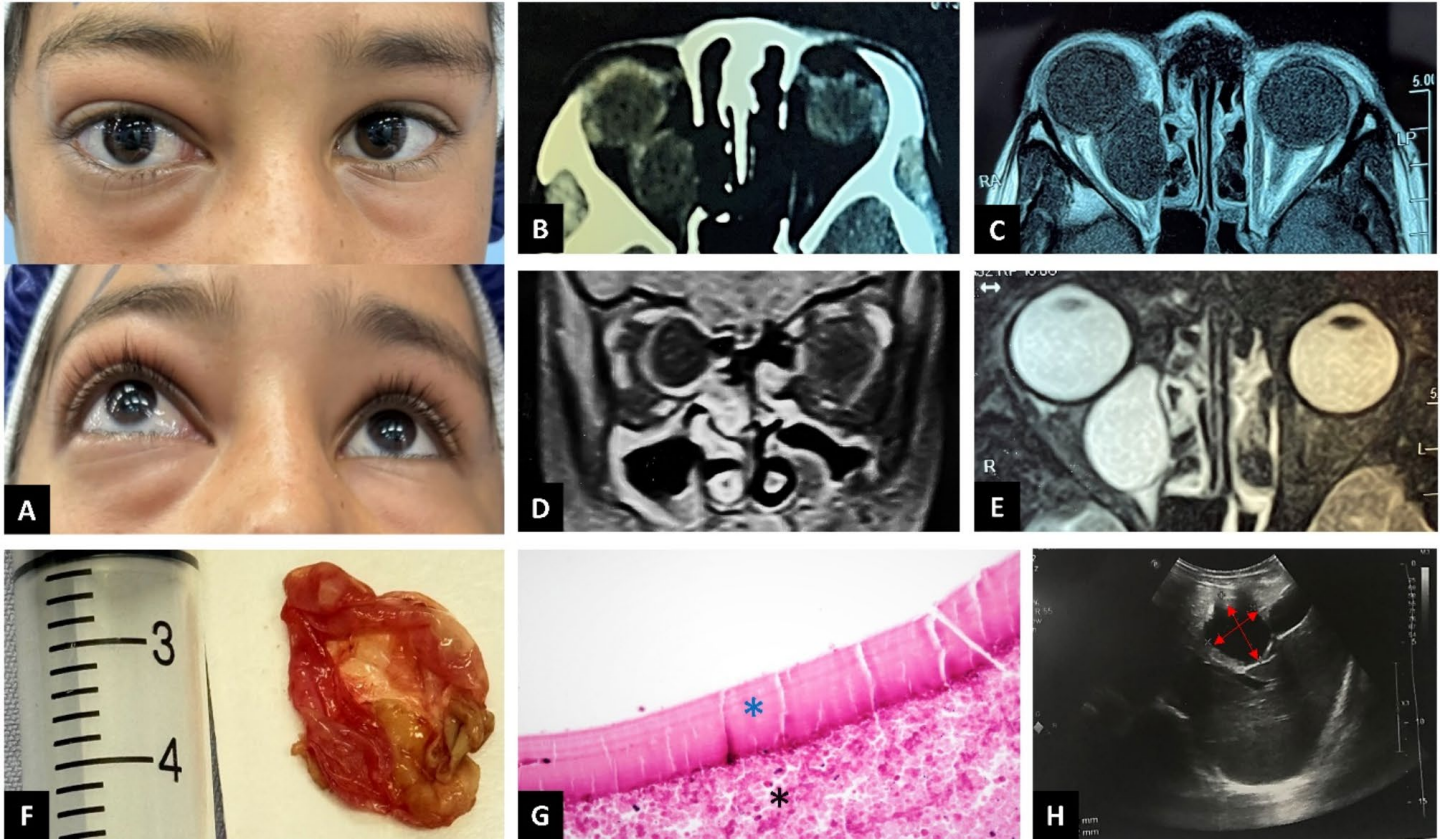
**A**: Proptosis with mild periocular swelling in the right eye. **B**: Axial view of an orbital computed tomography (CT) scan without contrast, showing a well-defined, round intraconal lesion without signs of bony erosion or remodeling. **C**: Corresponding axial view of T1-weighted orbital magnetic resonance imaging (MRI) without contrast, demonstrating the well-defined hypointense cystic lesion. **D**: Coronal view of T1-weighted MRI with contrast, showing the wall enhancement of the lesion without intralesional enhancement. **E**: Axial view of T2-weighted orbital MRI, showing a homogeneous cystic mass lesion hyperintense to the extraocular muscles and brain parenchyma. **F**: The walls of the excised cystic lesion are shown. **G**: Histopathological evaluation revealed the outer wall of the cyst as an acellular laminated membrane with internal germline epithelium (blue asterisk), surrounded by acute inflammatory cells and necrotic material (black asterisk) (hematoxylin & eosin staining, ×400 magnification). **H**: Liver ultrasonography revealing a cystic lesion measuring 35 × 50 mm (red double-headed arrows) in the left hepatic lobe

A 12-year-old boy from a rural area in Afghanistan, with exposure to wildlife dogs, presented to the oculoplastic clinic at Farabi Eye Hospital, Tehran, Iran, with a two-month history of progressive periocular swelling and globe protrusion on the right side (Fig. 1A). On ophthalmic examination, his best-corrected visual acuity was 20/20 in both eyes, with no relative afferent pupillary defect. Marked axial proptosis was noted in the right eye, measured at 22 mm on Hertel’s exophthalmometry, compared to 19 mm on the left. Periorbital swelling was also observed on the right side. Medial ocular motility of the affected side was limited to -3, while lateral motility was restricted to -1. The anterior and posterior segment examinations were unremarkable. Given the proptosis, orbital imaging was requested.

Orbital computed tomography (CT) revealed a medial orbital mass lesion isodense with the vitreous cavity, which was consistent with a cystic lesion (Fig. 1B). Orbital magnetic resonance imaging (MRI) revealed a large mass within the medial rectus muscle, extending posteriorly near the superior orbital fissure. On T1-weighted MRI, the mass appeared hypointense (Fig. 1C), with no enhancement within the lesion, but ring enhancement was observed (Fig. 1D). The mass showed homogeneous hyperintensity on T2-weighted imaging (Fig. 1E). These imaging characteristics were suggestive of an orbital cystic lesion.

Consequently, orbital surgery was planned to excise the mass. The approach was through the medial retro-caruncular plane. The cyst, located posteriorly within the medial rectus, was large and difficult to access, surrounded by thick fibrotic tissue. Dissection of the fibrotic tissues was attempted, followed by puncturing the cyst. Approximately 3 cc of serous fluid was aspirated from the cyst. The cyst was then irrigated with hypertonic saline, and its walls were gently dissected from the fibers of the medial rectus muscle and completely removed (Fig. 1F). Due to the cyst fluid leakage, irrigation was performed again with hypertonic saline to prevent larval seeding.

Histopathological examination of the cyst confirmed the structure of a hydatid cyst, with an acellular laminated membrane and germinal epithelium. Therefore, we decided to further investigate the patient for potential systemic involvement of the disease (Fig. 1G). A chest CT scan and abdominal ultrasonography, along with liver function tests, were requested. The chest CT was unremarkable, but abdominal ultrasonography revealed a large cyst (35 × 50 mm) in the left hepatic lobe, characteristic of a hydatid cyst (Fig. 1H). Additionally, alkaline phosphatase levels were elevated.

**Fig. 2:**
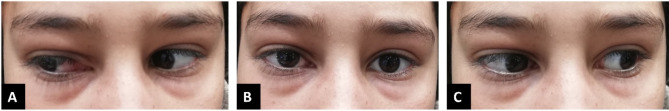
**A-C**: Two months after surgical excision of the cystic lesion in the right orbit, the proptosis had significantly regressed, with no obvious periocular or ocular surface inflammatory signs. Furthermore, the eyes were orthotropic, and the movements of the horizontal extraocular muscles were within normal limits

Given the diagnosis of hydatid disease, the patient was referred to an infectious disease specialist. Systemic treatment with oral Albendazole (400 mg twice daily) was initiated for one month. Two months after the surgical excision and adjuvant antiparasitic treatment, the patient showed significant improvement in periorbital swelling and proptosis, with no limitation in ocular motility (Fig. 2A-C). The patient was referred to a general surgeon for further evaluation and management of the hepatic cyst. No recurrence was observed at a six-month follow-up.

## Discussion

Hydatid disease is a common zoonotic infection, particularly in regions where traditional livestock farming is widespread. The two main species responsible are *Echinococcus granulosus* and *Echinococcus multilocularis* [10]. This condition, also known as cystic echinococcosis, is endemic in regions where livestock farming is prevalent and involves a life cycle with dogs, sheep, cattle, and goats. Endemic regions include Africa, Asia, Australia, and Mediterranean countries [10, 11]. Humans are accidental intermediate hosts, infected through ingestion of eggs from contaminated water, food, or direct contact with infected animals. Once ingested, the eggs hatch in the intestine, releasing oncospheres that migrate through the bloodstream to organs such as the liver and lungs, where they develop into fluid-filled cysts. Hydatid cysts occur most commonly in the liver (50–70% of cases) and less commonly in the lung, spleen, kidneys, and brain [2–4]. Although the liver is the most common primary organ affected, other organs may also be involved, either secondarily or in isolation. Previous literature has shown that the orbit, along with the brain and heart, can also be affected. Therefore, in any case suspected of hydatid cyst involvement in an organ (including the orbital cavity), a systemic workup for other organ involvement, particularly the liver, is both logical and necessary. In a few cases, orbital involvement has been the initial presentation of systemic hydatid disease, similar to our case [12–14]. Our patient initially presented with orbital symptoms and signs, without experiencing abdominal pain or other symptoms commonly associated with liver hydatid cysts. However, systemic evaluation revealed a large hepatic cyst.

Orbital hydatid cysts are most commonly seen in children and young adults, typically located in the retrobulbar region, either extraconally or intraconally [15]. According to previous studies, intraconal and superior locations are the most frequently reported sites for orbital hydatid cysts [16]. However, hydatid cysts within extraocular muscles have also been documented, likely due to the rich blood supply of these muscles [8, 17, 18]. In cases of intramuscular orbital hydatid cysts, the inferior and medial rectus muscles are the most commonly involved [8, 18–23]. The presentation of hydatid disease is primarily due to the mass effect of the cysts. The orbit is a confined space, so proptosis is the most common presentation of an orbital hydatid cyst [7]. Other orbital presentations may include visual impairment due to optic nerve compression, limitation of ocular motion due to extraocular muscle involvement, and periocular swelling or chemosis [1, 24]. The diagnosis of hydatid disease, including suspected orbital involvement, is typically made using imaging techniques such as ultrasonography, CT scan, and MRI [8]. Hydatid cysts typically appear as cystic fluid with water attenuation on CT scans. If the cyst rim is calcified, it may present as a hyperdense rim around the cyst. On MRI, hydatid cysts are hypointense on T1-weighted images and hyperintense on T2-weighted images, with a low-signal intensity rim on T2-weighted images. The rim may show enhancement following contrast injection. If daughter cysts are present, they can be observed as small cystic structures attached to the internal layer of the main cyst. These daughter cysts are hypointense relative to the intracystic fluid on T1-weighted images and hyperintense on T2-weighted images [25]. For further diagnostic confirmation, serological tests like enzyme-linked immunosorbent assay (ELISA) or Western blot may be useful, especially in uncertain cases. However, the role of biological tests is limited due to high rates of negative results, particularly in intact cysts. Eosinophilia and elevated erythrocyte sedimentation rate (ESR) may be observed in ruptured cysts but are nonspecific [26]. Histopathological evaluations of these cysts reveal three distinct layers: (a) the outer pericyst, composed of modified host cells that form a dense, fibrous protective layer; (b) the middle acellular laminated membrane; and (c) the inner germinal layer, where scolices (the larval stage of the parasite) and the laminated membrane are produced. Additionally, daughter cysts (brood capsules) may be present within the main cyst [25]. In our case, the patient was from a rural area in Afghanistan and had exposure to wildlife dogs. He initially presented with gradually increasing proptosis and periocular swelling. Orbital CT and MRI revealed a mass lesion consistent with a cyst involving the orbital cavity and the medial rectus muscle. Histopathological evaluation revealed a layered cyst, with an outer wall composed of an acellular laminated membrane and internal germinal epithelium, surrounded by acute inflammatory cells and necrotic material. A systemic evaluation, including liver sonography, revealed a liver cyst. Based on these findings, the patient was diagnosed with hydatid disease involving the orbital cavity.

Common differential diagnoses for orbital hydatid cysts include orbital cysticercosis, dermoid cysts, orbital abscesses, mucoceles, and hematocysts [12]. Among the differential diagnoses, orbital cysticercosis is a particularly similar condition caused by the larval form of Taenia solium. It can present as a cystic orbital lesion with inflammatory or mass effect symptoms affecting various orbital compartments, including extraocular muscles, making differentiation from hydatid cysts challenging based solely on clinical and imaging findings [27]. However, a history of potential exposure, consideration of endemic regions, and laboratory tests (e.g., ELISA or Western blot) can aid in differentiation. Definitive diagnosis requires surgical excision and histopathological evaluation [28].

The treatment of choice for a hydatid cyst is surgical excision. Some investigators suggest preoperative administration of antiparasitic drugs to prevent parasite seeding and reduce the risk of anaphylactic reactions in cases where the cyst may rupture during surgery [26, 29]. In cases with orbital involvement, surgery should be performed as soon as possible, particularly when cyst-induced compressive effects, severe proptosis, or exposure keratopathy are present [20]. Complete resection of an orbital cyst without rupture is often difficult due to the limited orbital space. As mentioned previously, the hydatid cyst consists of three layers, with the external layer forming a fibrous capsule that surrounds the other two layers and adheres to orbital structures, making dissection challenging [30]. Therefore, surgical excision of these lesions is difficult, as encountered during the operation in our case. Choosing a safe surgical approach depends on the location of the orbital hydatid cyst. Rajabi et al. reported eight cases of orbital hydatid cysts at various orbital locations and used lateral, superior, and medial approaches for surgery [20]. Attar et al. removed a medial rectus cyst by excising the lateral orbital wall to improve access, instead of performing a medial orbitotomy due to the confined space in the medial orbit [17]. In our case, we approached the cyst through the retro-caruncular plane. For a large cyst within the inferior rectus, Haydar et al. and Ghaedamini et al. performed an inferior trans-conjunctival orbitotomy [8, 18]. Hammoud et al. used a fronto-orbital approach to excise a large intraconal cyst [31]. According to the literature, performing a puncture and aspiration first helps reduce the cyst size and causes the inner germinative layer to collapse, allowing the complete removal of the germinal endocyst [31]. Complete removal of an orbital hydatid cyst without rupture is unfeasible, making PAIR (puncture, aspiration, irrigation, and re-aspiration) the most effective surgical approach [32]. In our case, after puncture and aspiration, the cyst was irrigated with hypertonic saline and re-aspirated before the complete removal of the intramuscular cyst wall.

Following surgery, initiating systemic antiparasitic medication is crucial. Albendazole is the preferred choice due to its broad-spectrum antiparasitic activity, and in our case, it was prescribed for one month, resulting in improvement of the disease signs and symptoms [33]. In contrast, mebendazole has limited efficacy for intraorbital hydatid cysts due to its insufficient penetration into the orbit and poor ability to cross the blood-brain barrier [34].

In conclusion, although orbital hydatid cysts are rare, they should be considered in the differential diagnosis of orbital masses in endemic regions, especially in pediatric populations. Accurate diagnosis, appropriate surgical management, and thorough systemic evaluation are essential to prevent recurrence and damage to orbital structures.

## Data Availability

No datasets were generated or analysed during the current study.
